# Thermodynamic Properties of Stoichiometric Non-Superconducting Phase Y_2_BaCuO_5_

**DOI:** 10.3390/ma12193163

**Published:** 2019-09-27

**Authors:** Filip Antončík, David Sedmidubský, Adéla Jiříčková, Michal Lojka, Tomáš Hlásek, Květoslav Růžička, Ondřej Jankovský

**Affiliations:** 1 Department of Inorganic Chemistry, Faculty of Chemical Technology, University of Chemistry and Technology, Technická 5, 166 28 Prague, Czech Republic; Filip.Antoncik@vscht.cz (F.A.); Adela.Jirickova@vscht.cz (A.J.); michal.lojka@vscht.cz (M.L.); ondrej.jankovsky@vscht.cz (O.J.); 2 Can Superconductors, 251 68 Kamenice, Czech Republic; tomas.hlasek@cansuperconductors.com; 3 Department of Physical Chemistry, Faculty of Chemical Technology, University of Chemistry and Technology, Technická 5, 166 28 Prague, Czech Republic; ruzickak@vscht.cz

**Keywords:** Y-211, superconductors, Y_2_BaCuO_5_, thermodynamic properties, YBCO

## Abstract

Y_2_BaCuO_5_ often occurs as an accompanying phase of the well-known high-temperature superconductor YBa_2_Cu_3_O_7_ (also known as YBCO). Y_2_BaCuO_5_, easily identifiable due to its characteristic green coloration, is often referred to as ‘green phase’ or ‘Y-211’. In this contribution, Y_2_BaCuO_5_ phase was studied in detail with a focus on its thermal and thermodynamic properties. Energy dispersive spectroscopy (EDS), X-ray diffraction (XRD), and scanning electron microscopy (SEM) were employed in the study of sample’s morphology and chemical composition. XRD data were further analyzed and lattice parameters refined by Rietveld analysis. Simultaneous thermal analysis was employed to study thermal stability. Particle size distribution was analyzed by laser diffraction. Finally, thermodynamic properties, namely heat capacity and relative enthalpy, were measured by drop calorimetry, differential scanning calorimetry (DSC), and physical properties measurement system (PPMS). Enthalpy of formation was assessed from ab-initio DFT calculations.

## 1. Introduction

Transition metal (TM) oxides constitute a large family of oxides, which possess a variety of interesting properties [1,2,3,4]. Cobalt mixed oxides with misfit structure are thermoelectric materials for the recovery of waste heat, and misfit cobaltites also show unique catalytic properties [5,6,7]. Mixed oxides containing lithium found a crucial application in accumulator industry [8]. Nano-sized oxides are widely used in catalysis, electrochemistry, and solar cells [9,10,11,12,13]. Among TM oxides, the high-temperature superconductors (HTS) revealing critical temperature close to the temperature of liquid nitrogen have been attracting permanent attention since their discovery in 1986 [14]. Although enhanced critical temperatures and intriguing superconducting behavior have been later reported in several systems, for example in layered ternary and quaternary arsenide systems [15,16], the HTS cuprates became the most wide-spread group of high-temperature superconductors, still being at the forefront of both theoretical and applied research in the field of superconductivity. 

Their discovery in the Ba–La–Cu–O system [14] was immediately followed by an intensive search for novel HTS phases in congeners systems such as Y–Ba–Cu–O, Bi–Sr–Ca–Cu–O, Tl–Ba–Ca–Cu–O, and Hg–Ba–Ca–Cu–O [17,18,19,20,21,22,23,24,25]. Superconducting phases in these systems are usually derived from perovskites by inserting other structural motifs such as rock-salt type blocks, and thus forming layered structures. In general, perovskites and similar layered oxides exhibit a wide variety of interesting properties, such as high electrochemical stability, catalytic activity, low electric resistivity, and low thermal conductivity [26,27,28,29]. For the properties listed above, layered inorganic oxides are still studied extensively, even to this day. One of the most significant systems relevant to HTS is the quasi-ternary Y–Ba–Cu–O system [30], with YBa_2_Cu_3_O_7-δ_ (YBCO) being the superconductive phase [31]. This phase is usually accompanied by Y_2_BaCuO_5_ [32,33,34,35], which is often formed during the solid-state reaction [36], as a result of surplus of yttrium. This can be caused by partial melting during the processing yielding a Ba- and Cu-rich melt, which subsequently crystallizes into Ba_3_Cu_5_O_8_ phase during cooling if the equilibrium is not achieved. As a result, the rest of the system is shifted towards Y-rich compositions, inducing a formation of Y_2_BaCuO_5_. However, this effect is not necessarily detrimental for the superconductivity of the material, since Y_2_BaCuO_5_, if properly nanostructured, can act as a source of pinning centers for vortices. The goal of this paper is to study Y_2_BaCuO_5_ phase that is present in single-domain YBCO superconductors in a form of pinning centers. The main focus is put on the thermal properties, which are decisive for tailoring the material properties by high temperature processing. 

Similarly to YBa_2_Cu_3_O_7-δ_, copper is five-fold coordinated by oxygen atoms; however, the tetragonal pyramids are not interconnected, yielding an insulating ground state. Moreover, at temperatures below 30 K, an antiferromagnetic arrangement of spins ½ localized on Cu(+II) sites is established [37]. These physical characteristics have to be considered when interpreting the heat capacity at low temperatures.

In our contribution, we prepared pure polycrystalline phase Y_2_BaCuO_5_. Phase purity, morphology, and thermal and thermodynamic properties were studied in detail. The precise knowledge of thermodynamic properties of phases involved in the Y–Ba–Cu–O system is essential for modeling phase equilibria in YBCO-based superconducting materials (including systems with additional components) and tailoring their micro/nanostructural characteristics to enhance their performance.

## 2. Materials and Methods 

We used the following chemicals for the synthesis of Y_2_BaCuO_5_: Barium carbonate (Sigma-Aldrich, Prague, Czech Republic; 99 + %), yttrium (III) oxide (Chemapol, Prague, Czech Republic; 99.99%), and copper (II) oxide (Sigma-Aldrich; 99.99%). Powders were homogenized in the ratio corresponding to Y_2_BaCuO_x_ stoichiometry and repeatedly calcined in corundum crucible at 1123 K for 12 h, then at temperatures of 1138 K, 1153 K, and 1163 K for 24 h with a homogenization between each step. Finally, the sample was pressed into pellets and calcined for additional 24 h at 1173 K. The sample was slowly cooled in order to obtain a thermodynamically stable phase fully saturated with oxygen. The sample with the stoichiometry Y_2_BaCuO_5_ is termed Y-211 hereinafter.

X-ray powder diffraction (XRD) was measured at 298 K on Bruker D8 Discoverer powder diffractometer (Bruker, Karlsruhe, Germany) in a standard parafocusing Bragg–Brentano geometry using CuK_α_ radiation (*λ* = 1.5418 Å, *U* = 40 kV, *I* = 40 mA). Rietveld refinement fit was performed using Topas software v. 5 (Bruker, Karlsruhe, Germany).

The microstructure was analyzed using scanning electron microscopy (SEM). The elemental composition was studied using energy-dispersive spectroscopy (EDS) analyzer X-Max^N^ with 20 mm^2^ silicon drift detector from Oxford instruments (Abingdon, UK) and software package Aztec Energy v. 3 (Asylum Research-Nanoanalysis, Wycombe, UK) using a Tescan Lyra dual beam microscope (Tescan, Brno, Czech Republic) with a field emission gun electron source.

The thermal stability Y_2_BaCuO_5_ was analyzed by simultaneous thermal analysis (STA) using Setaram Setsys Evolution (Setaram, Lyon, France). The heating rate during the measurement performed in dynamic air atmosphere (50 cm^3^ min^−1^) was 10 K min^−1^.

The particle size distribution was analyzed by the laser diffraction method; Malvern Panalytical Mastersizer 3000 device (Malvern, UK) with 4 mW He–Ne 632.8 nm Red light source and 10 mW LED 470 nm Blue light source was used. The range of measurement was set from 0.1 to 1000 μm. The measurement was done using dispersion (1 g/100 mL) of the material in isopropyl alcohol. Y-211 sample was measured 5 times (5 scans) and distribution was created as an average value.

The heat capacity in the low-temperature region was measured using the physical properties measurement system (PPMS) equipment, Evercool-II 9 T-type (Quantum Design, San Diego, CA, USA). The heat capacity was obtained by the relaxation method under high vacuum. 

A Tian–Calvet-type calorimeter μDSC IIIa (Setaram, Lyon, France) was used for the measurement of heat capacities in the temperature range 262 to 358 K, employing NIST Standard reference material No. 720 as a reference material. Performance of the calorimeter is regularly checked by measurement of compounds with well-established *C_pm_* data (naphthalene, benzophenone, benzoic acid). Based on these checking experiments, the combined expanded uncertainty of the heat capacity measurements is estimated to be *U*_c_(*C_p_*_m_) = 0.01 *C_p_*_m_. More details can be found in paper [38]. Data obtained from this calorimeter are referred as DSC data.

Multi HTC 96 (Setaram, Lyon, France) high-temperature calorimeter (operated in a static air atmosphere) was used to measure enthalpy increments. The measurement was performed by dropping of the reference material (synthetic sapphire, NIST No. 720) and the sample in the sequence standard–sample–standard–sample–standard. The delays between two subsequent drops were 25 min in order to stabilize the heat flow. The estimated combined expanded uncertainty of the heat capacity measurements is estimated to be *U*_c_(*C_p_*_m_) = 0.03 *C_p_*_m_.

Enthalpy of formation was assessed from ab-initio density functional theory (DFT)-based calculations. The electronic structure and total energies of Y_2_BaCuO_5_ and the constituent oxides, Y_2_O_3_, BaO, and CuO, were calculated by means MedeA-VASP [39] program using projector augmented plane waves basis set and generalized gradient approximation (GGA-PBE0 [40]) to exchange-correlation functional combined with additional local orbital specific coulomb potential *U* = 3 eV acting on Cu-3d states [41]. An antiferromagnetic ground state was considered for both CuO [42] and Y_2_BaCuO_5_ [37]. The basis set with a cut-off energy of 400 eV was applied. The k-point mesh was constructed inside the first Brillouin zone with k-point spacing smaller than 0.25 Å^−1^. A tetrahedron integration scheme was applied for electron density of states calculation.

## 3. Results and Discussion

**Figure 1:**
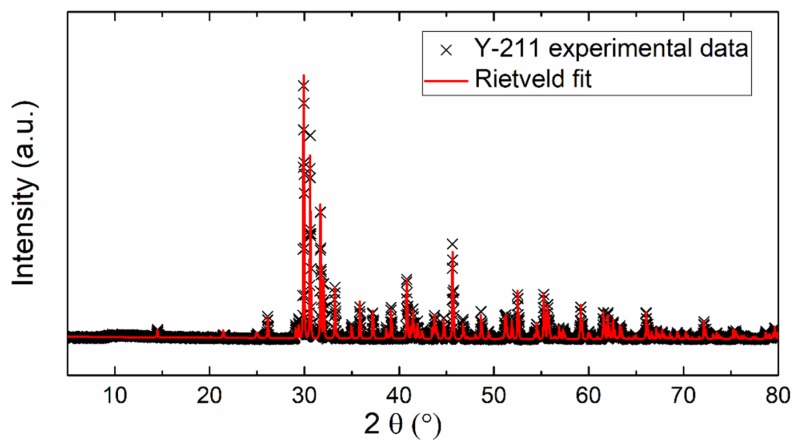
XRD powder diffractogram and Rietveld fit of experimental data.

Figure 1 shows the XRD pattern of the prepared Y_2_BaCuO_5_ sample. No visible impurities were detected by X-ray diffraction, only the presence of the Y-211 phase (Y_2_BaCuO_5_). The Rietveld fit (based on the orthorhombic space group *Pnma*) was applied to refine the lattice parameters. The following lattice parameters were obtained: *a* = 12.134 Å, *b* = 5.6614 Å, and *c* = 7.1347 Å (*α* = *β* = *γ* = 90° being fixed). The obtained data are in good agreement with the already published results *a* = 12.1630 Å, *b* = 5.6490 Å, and *c* = 7.1230 Å (JCPDS 01-078-2214).

**Figure 2:**
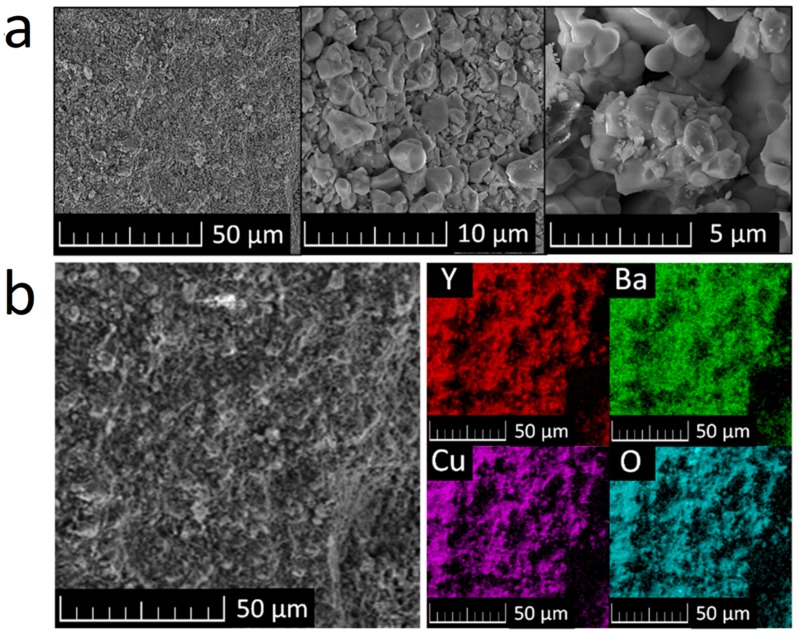
Y_2_BaCuO_5_ SEM micrographs (**a**) and elemental distribution maps of obtained by EDS (**b**).

SEM micrographs of the sample show that the Y_2_BaCuO_5_ particle size lies mostly between 1 and 5 μm; the particle size remained very homogeneous across the sample, as can be seen from Figure 2a. This is in good agreement with the microstructures typically observed in polycrystalline cuprates. Elemental distribution maps were obtained by EDS and can be seen in Figure 2b. The sample appears to be homogenous and pure from a chemical point of view. Only Y, Ba, Cu, and O were detected. These results correspond with the results obtained by X-ray diffraction and confirm purity and phase composition of the prepared sample.

**Figure 3:**
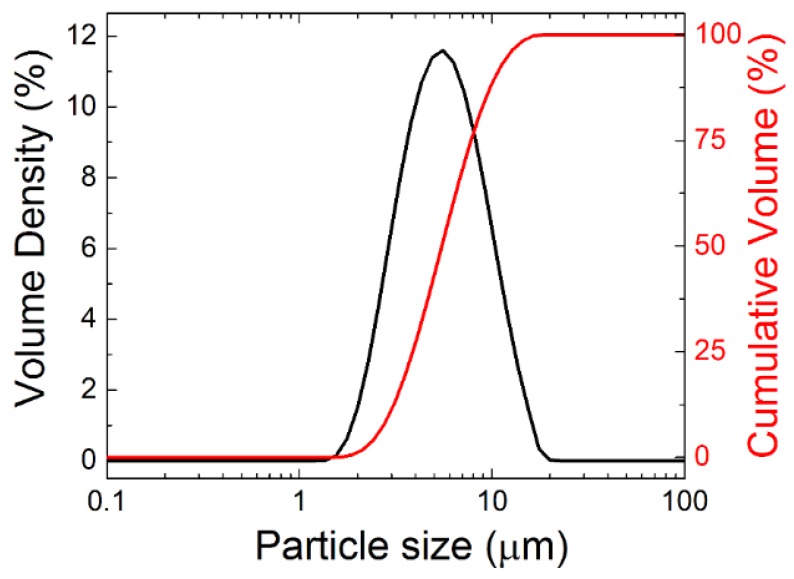
Particle size distribution of Y_2_BaCuO_5_ powder using laser diffraction.

In order to confirm the sizes of grains obtained from SEM, the powder was also analyzed by laser diffraction. Particle size distribution is shown in Figure 3, revealing that the grains are mainly of the size between 1 and 10 μm, dx(50) = 5.2 μm, dx(10) = 2.8 μm and dx(90) = 10.3 μm. These data are in good agreement with SEM.

**Figure 4:**
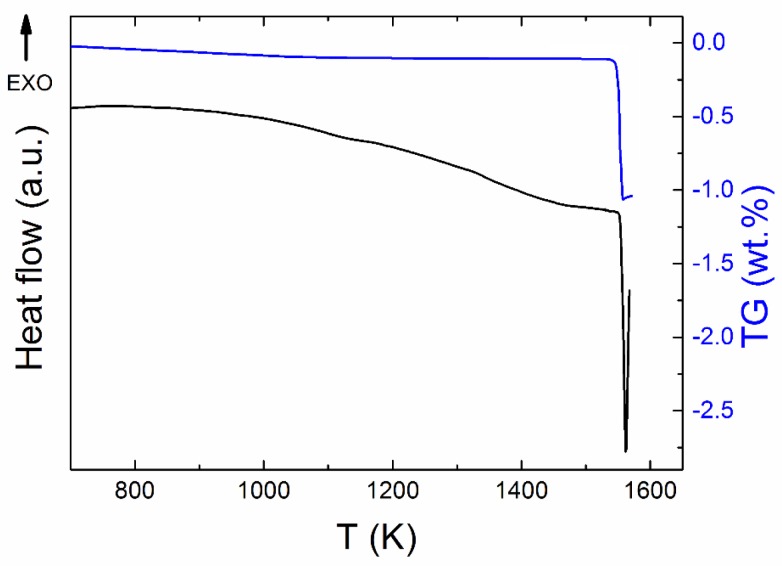
STA measurement of Y_2_BaCuO_5_ in dynamic air atmosphere.

Simultaneous thermal analysis (STA) was employed to study the thermal behavior of Y_2_BaCuO_5_ (Figure 4). The phase Y-211 decomposed in air atmosphere at 1551 K. Throughout the measurement, the mass was almost constant, i.e., the phase was stable up to temperatures of ~1550 K and we did not detect any release of oxygen, suggesting the presence of the stoichiometric phase. The weight decrease related to the original mass of Y_2_BaCuO_5_ was approximately 1 wt % suggesting formation of Y_2_O_3_ (s), BaO (s), and Cu–O (l).

**Figure 5:**
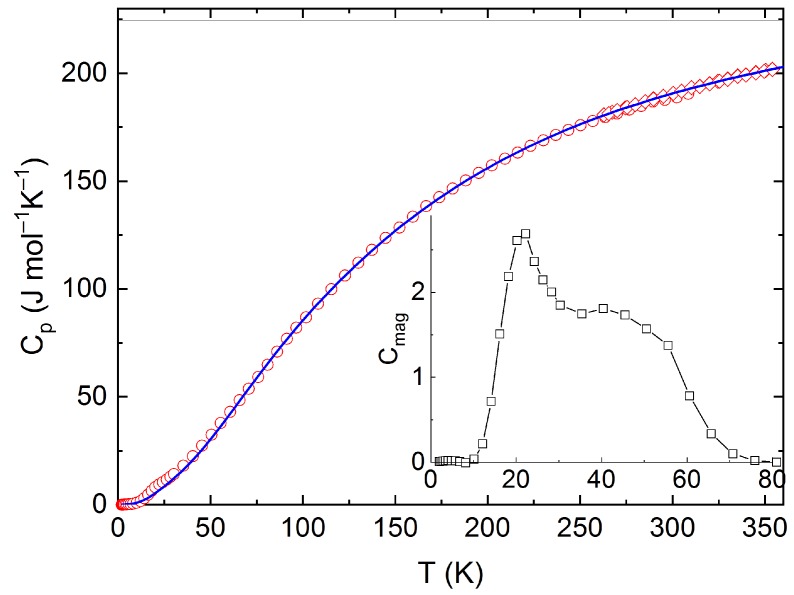
Low temperature heat capacity of Y_2_BaCuO_5_ obtained from PPMS and DSC, including the Debye–Einstein fit and the excess magnetic term in the inset.

**Figure 6:**
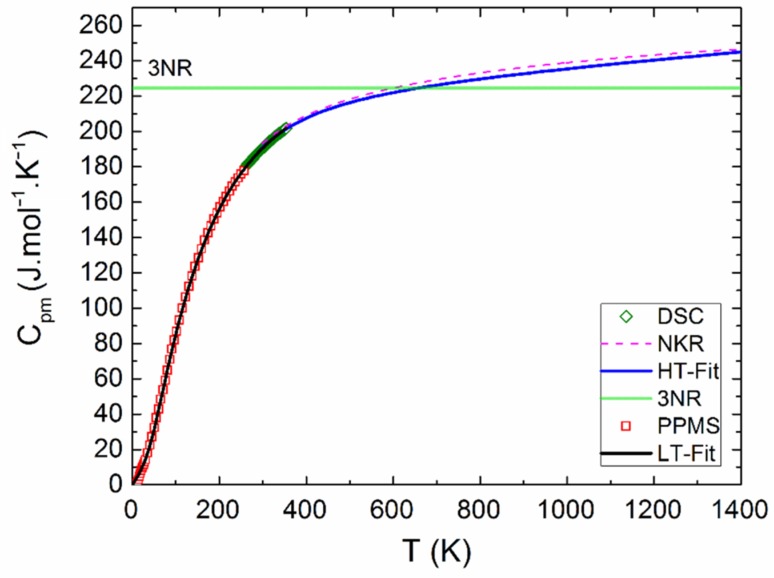
Heat capacity of Y_2_BaCuO_5_ obtained from PPMS and HT-fit.

**Figure 7:**
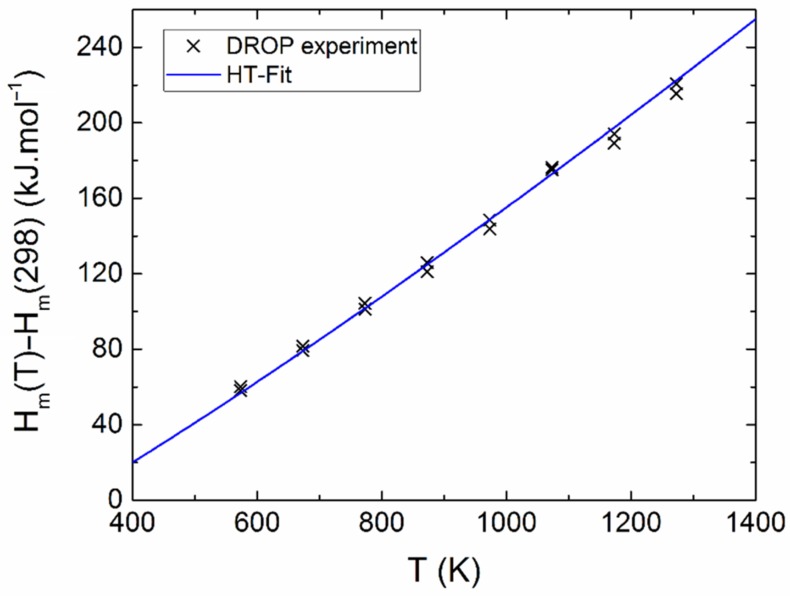
Relative enthalpy of Y_2_BaCuO_5_ obtained from drop experiments.

The measured calorimetric data used for further analysis involved 37 *C_p_*_m_ values from relaxation time method (30–257 K), 40 *C_p_*_m_ values from DSC (263–354 K) (see Figure 5 and Figure 6), and 16 values of the enthalpy increments from the drop measurements from the range 573–1273 K (see Figure 7).

For further analysis of low temperature heat capacity data, two sets of the *C_pm_* data (relaxation time + DSC) were considered. Since the studied material is a magnetic insulator, only the lattice part of heat capacity is relevant for temperatures well above the Néel temperature, *T* = 16.2 K. For this reason, only the data above 30 K were considered for the analysis of phonon heat capacity, which was performed using the combination of the Debye and Einstein models according to the following Equation (1):

(1) $$C_{phD}=R\left[9{\left(\frac{T}{\Theta_{D}}\right)}^{3}\int_{0}^{x_{D}}\frac{x_{D^{4}}exp\left(x_{D}\right)}{{\left(exp\left(x_{D}\right)-1\right)}^{2}}dx+\overset{3}{\underset{i=1}{\sum }}\frac{w_{i}x_{Ei}^{2}exp\left(x_{Ei}\right)}{{\left(exp\left(x_{Ei}\right)-1\right)}^{2}}\right]$$

Here *R* is the gas constant, *θ_D_* and *θ_Ei_* are the Debye and Einstein characteristic temperatures, and *x_D_* = *θ_D_*/*T*, *x_Ei_* = *θ_Ei_*/*T,* and *w_i_* refer to a degeneracy of the corresponding Einstein mode. 

It is a well-known fact that the phonon spectrum of a mixed oxide contains 3*n*–3 optical branches and three acoustic branches (*n* is number of atoms per formula unit). In Y_2_BaCuO_5_, there are 9 atoms per formula unit representing 24 optical branches which were grouped into three 8-fold degenerate Einstein modes. 

The Debye–Einstein model is based on a harmonic crystal approximation describing the heat capacity at constant volume. As can be seen Figure 6, the experimentally obtained heat capacity exceeded the Dulong–Petit limit valid for *C_V_* at ~670 K. It was previously described [43] that a multiplication factor 1/(1−*αT*) can be introduced to any vibration mode (in order to take into account the anharmonic effects) and to describe the *C_p_–C_V_* difference using a semi-empirical approach. Hence, in our analysis, we used such an anharmonicity parameter that was combined only with the Debye mode in Equation (1). The analysis of the phonon heat capacity is shown in Table 1. The fitted curve based on PPMS and DSC data corresponding to LT-fit in Figure 5 describes the low temperature data satisfactorily above 60 K. Below this temperature, a substantial excess term due to magnetic ordering is clearly visible (see the inset in Figure 5).

The absolute entropy at the reference temperature (*T* = 298.15 K) was obtained by integrating the experimental data of the heat capacity divided by the thermodynamic temperature from 2 K up to ambient temperature as *S_m_*(298.15) = 209.47 J mol^−1^ K^−1^. This experimental value can be decomposed into a lattice vibrations contribution, *S_lat_*(298.15) = 206.9 J mol^−1^ K^−1^, and an excess entropy due to magnetic ordering disruption *S_lat_*(298.15) = 2.8 J mol^−1^ K^−1^, which is about two times lower than the theoretical value *R* ln2 corresponding to spin ½ of Cu(+II) and also lower than the experimental value reported by Knafo et al. [43] who, however, derived the phonon contribution by scaling the *C_p_* data of YBa_2_Cu_3_O_7_.

Above room temperature, dependence of the heat capacity was also determined. The linear least-squares method was used for treatment of the heat capacity data from DSC and the enthalpy increment data from drop calorimetry. The temperature dependence of *C_pm_* was obtained in Equation (2):

(2) $$C_{pm}=A+B\times T+C\times T^{-2}$$

Let us note that the related temperature function of enthalpy, Δ*H*_m_(*T*) = *H*_m_(*T*) − *H*_m_(*T*_0_), is given by the following Equation (3):

(3) $$\Delta H\left(T\right)=H\left(T\right)-H\left(T_{0}\right)=A\times \left(T-T_{0}\right)+\frac{1}{2}B\times \left(T^{2}-T_{0}^{2}\right)-C\times \left(\frac{1}{T}-\frac{1}{T_{0}}\right)$$

The sum of squares was applied on both functions, Equations (2) and (3), and both sets of data were simultaneously minimized. Different weights *w_i_* were assigned to the individual points (calculated as *w_i_* = 1/*δ_i_*, where *δ_i_* is the absolute error bar). The obtained temperature dependence of heat capacity can be expressed as (valid between 298 and 1400 K) Equation (4):

(4) $$C_{pm}=\left(201.776\pm 10.531\right)+\left(0.041175\pm 0.016247\right)\times T-\left(2.21863\pm 0.3590\right)\times 10^{6}\times T^{-2}$$

We compared obtained data with the curve calculated using the Neumann–Kopp rule (NKR), where the heat capacity of Y_2_BaCuO_5_ was evaluated by the following Equation (5):

(5) $$C_{pm}\left(Y_{2}{\mathrm{BaCuO}}_{5}\right)=C_{pm}\left(Y_{2}O_{3}\right)+C_{pm}\left(\mathrm{BaO}\right)+C_{pm}\left(\mathrm{CuO}\right)$$

As can be seen from Figure 6, our data are in good agreement with the calculated NKR curve, suggesting that the lattice dynamics—including the anharmonic effects—are comparable in Y_2_BaCuO_5_ and the constituent binary oxides.

We took advantage of this similarity in chemical bonding and used the binary oxides as a reference for evaluating the enthalpy of formation from GGA+U ab-initio calculations. Hence, the ground state enthalpy of formation from oxides, Δ_ox_*H* = −32.2 kJ mol^−1^, is the primary value derived from DFT while the enthalpy of formation from elements, Δ_f_*H* = −2640.9 kJ mol^−1^, was obtained from Δ_ox_*H* and the tabulated Δ_f_*H* values of the binary oxides. The enthalpy of formation is underestimated compared to dissolution calorimetry results Δ_f_*H* = −2656 kJ mol^−1^ [44] and −2660 kJ mol^−1^ [45]. A part of this discrepancy can by accounted for by the negative integral of Δ_ox_*C*_p_ used to recalculate the ab-initio value from 0 to 298 K, for which the experimental data are reported.

**Figure 8:**
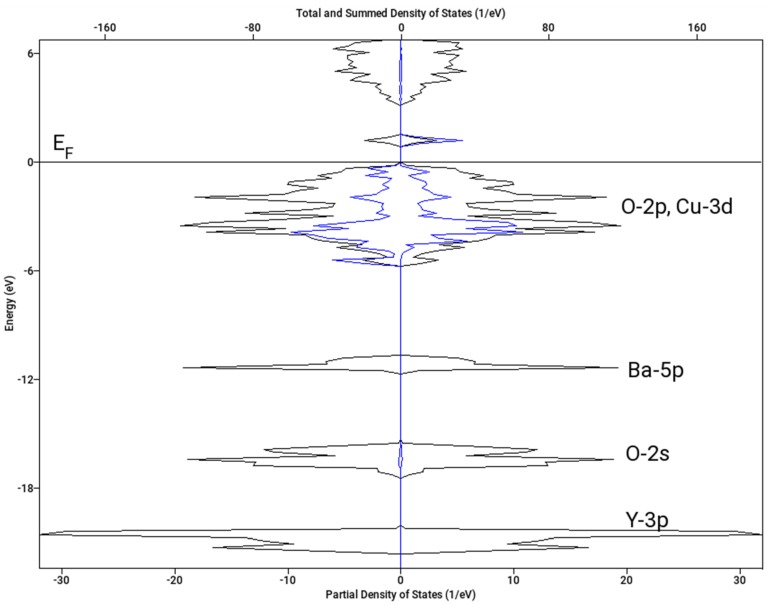
Calculated density of states (DOS) of Y_2_BaCuO_5_ (A-type antiferromagnetic arrangement). Spin-down projections is shown as negative. Total DOS—black line; Cu-3d projection for Cu(1) atom with down majority spin—blue line. Fermi level (*E*_F_) is set to 0 eV.

Let us note that the magnetic structure has a substantial effect on the resulting value of Δ_ox_*H*. Here, we present the results for A-type spin arrangement (two spins +½ and two spins −½ within the chemical unit cell) and no magnetic superstructure. The resulting density of states for both spin channels is shown in Figure 8. The spin polarization on copper sites clearly visible on Cu(1)-3d projection in Figure 8 amounts to 0.59 Bohr magnetons, which is in nice agreement with the neutron diffraction data (0.55 μB) [37]. If we considered the F type arrangement (all four spins aligned parallel) in the chemical unit cell and a magnetic superstructure with the wave vector *k* = [0, ½, ½], which has been reported as an alternative ordering, the calculation converged to a non-spin polarized state and Δ_ox_*H* = −0.5 kJ mol^−1^. Interestingly, a similar result (zero spin and low stabilization) was obtained for A-type ordering and *k* = [0, ½, ½] superstructure. Hence, the magnetic superstructure reported in [37] was not confirmed.

## 4. Conclusions

In our contribution, we prepared a polycrystalline cuprate Y_2_BaCuO_5_ by conventional solid-state reaction. The sample was thoroughly analyzed in order to ensure its purity. Morphology and thermal properties were further studied in detail. Using X-ray diffraction, we confirmed the presence of pure Y_2_BaCuO_5_ without any detectable impurities. Very high thermal stability (up to ~1550 K) of Y_2_BaCuO_5_ (compared to phase YBa_2_Cu_3_O_7_) is important for its inert behavior during the manufacturing of YBCO bulk ceramics. Moreover, the obtained calorimetry data are important for thermodynamic modeling of high temperature material processing, which can underpin future development of high-temperature superconductors and their possible applications in superconducting bearings, transport, or superconducting transmission lines.

## Figures and Tables

**Table 1:** Evaluated parameters of Debye–Einstein model based on PPMS and DSC data.

Mode (*i*)	*D*	*E*_1_	*E*_2_	*E*_3_	*_D_*
*w_i_*	3	8	8	8	-
*θ*_i_/K	116.3 ± 1.7	263.6 ± 2.0	406.7 ± 3.0	774.0 ± 3.9	(6.584 ± 0.120) × 10^−4^
